# Exploring mechanisms of skin aging: insights for clinical treatment

**DOI:** 10.3389/fimmu.2024.1421858

**Published:** 2024-11-08

**Authors:** Meiqi Zhang, Yumeng Lin, Zhongyu Han, Xuewen Huang, Shuwei Zhou, Siyu Wang, Yan Zhou, Xuan Han, Haoran Chen

**Affiliations:** ^1^ School of Medical and Life Sciences, Chengdu University of Traditional Chinese Medicine, Chengdu, China; ^2^ Health Management Center, Nanjing Tongren Hospital, School of Medicine, Southeast University, Nanjing, China; ^3^ Zhongda Hospital, School of Medicine, Southeast University, Nanjing, China; ^4^ Science and Education Department, Chengdu Xinhua Hospital Affiliated to North Sichuan Medical College, Chengdu, China; ^5^ Jiangsu Key Laboratory of Molecular and Functional Imaging, Department of Radiology, Zhongda Hospital, School of Medicine, Southeast University, Nanjing, China; ^6^ Department of Gastroenterology, The First Hospital of Hunan University of Chinese Medicine, Changsha, China; ^7^ Department of Dermatology, Guangzhou Dermatology Hospital, Guangzhou, China; ^8^ First Clinical College of Changzhi Medical College, Changzhi, China

**Keywords:** aging, type 2 inflammatory, chronic pigmentary disorders, skin cancer, skin

## Abstract

The skin is the largest organ in the human body and is made up of various cells and structures. Over time, the skin will age, which is not only influenced by internal factors, but also by external environmental factors, especially ultraviolet radiation. Aging causes immune system weakening in the elderly, which makes them more susceptible to dermatosis, such as type 2 inflammatory mediated pruritus. The immune response in this condition is marked by senescent cells consistently releasing low amounts of pro-inflammatory cytokines through a senescence-associated secretory phenotype (SASP). This continuous inflammation may accelerate immune system aging and establish a connection between immune aging and type 2 inflammatory skin diseases. In addition, two chronic pigmentation disorders, vitiligo and chloasma, are also associated with skin aging. Aged cells escape the immune system and accumulate in tissues, forming a microenvironment that promotes cancer. At the same time, “photoaging” caused by excessive exposure to ultraviolet radiation is also an important cause of skin cancer. This manuscript describes the possible links between skin aging and type 2 inflammation, chronic pigmentation disorders, and skin cancer and suggests some treatment options.

## Introduction

**Figure 1:**
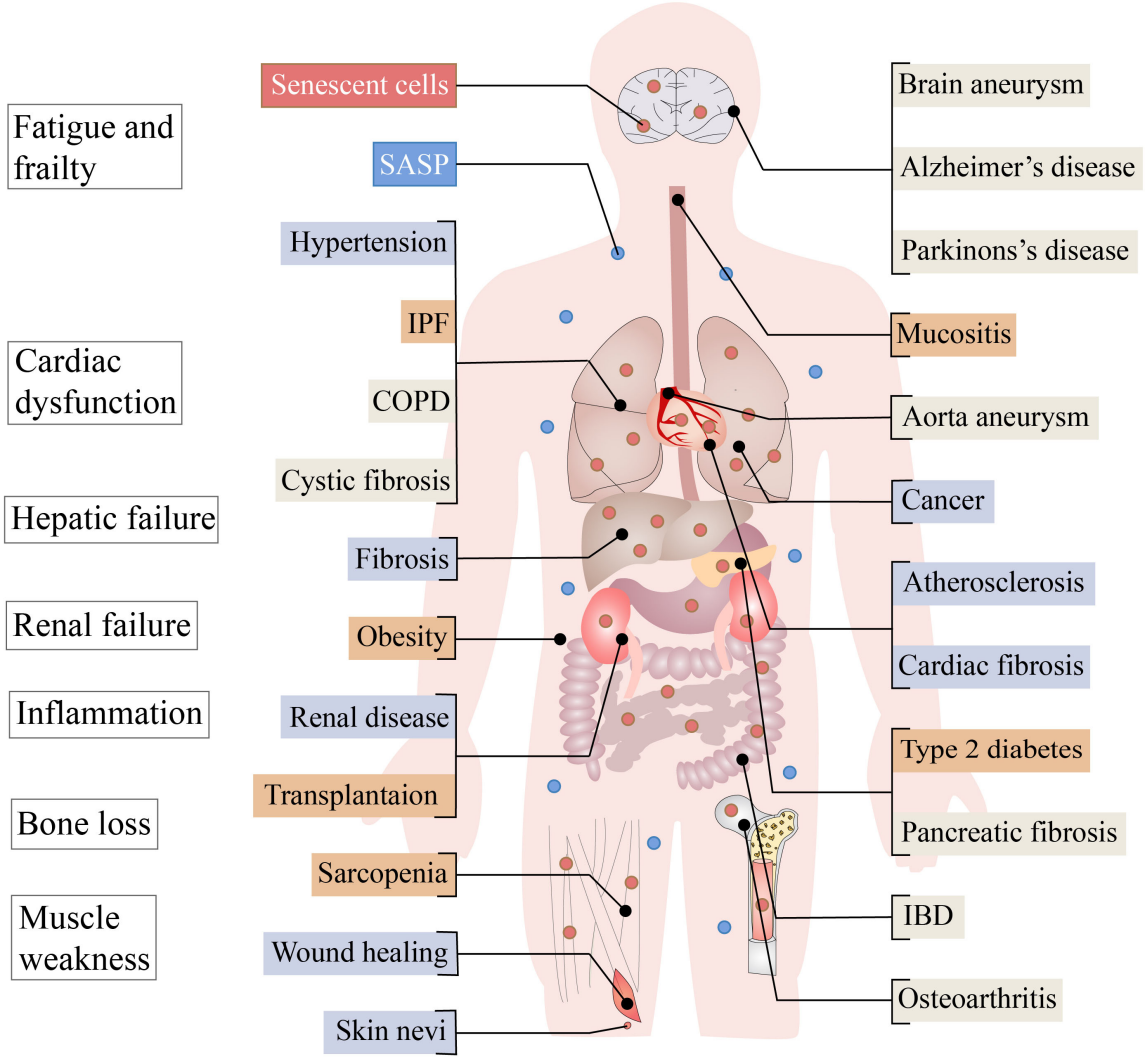
Aging in adult organ tissues. Senescent cells and associated harmful SASP can cause problems such as fatigue and frailty, cardiac dysfunction, hepatic failure, renal failure, inflammation, bone loss and muscle weakness. Diseases for which the beneficial or harmful effects of aging have not been determined are represented by beige boxes, while the known benefits of aging are represented by blue boxes and the harmful by red boxes. COPD, chronic obstructive pulmonary disease; IBD, inflammatory bowel disease; IPF, idiopathic pulmonary fibrosis; OSMF, oral submucous fibrosis; SASP, senescence-associated secretory phenotype.

The aging process affects not only the skin but also various tissues and organs of the human body (**Figure 1**). The skin, a vital and dynamic organ, serves as a crucial shield against harsh external conditions. It defends against dryness, chemical harm, and low body temperature, while also protecting the body against harmful pathogens through a multifaceted innate immune response and microbiome (1). As the skin barrier ages, skin immunosenescence occurs naturally, leading to a higher occurrence of skin diseases and more severe forms of these diseases in older individuals.

The elderly population is particularly affected by type 2 inflammatory skin diseases, such as atopic dermatitis (AD), chronic spontaneous urticaria (CSU), and bullous pemphigoid (BP), which are believed to be linked to skin immunosenescence (2). Additionally, vitiligo and chloasma, two contrasting pigmentation disorders, are associated with skin aging involving the dermal region and its paracrine function (3). It is well established that senescent cells persist over time and promote the proliferation of tumor cells. In a controlled environment, senescent fibroblasts also promote the growth of precancerous epithelium and give rise to tumor cells (4). Furthermore, weakened barrier function due to altered immune systems in older adults makes them more susceptible to cancer (5). Besides, DNA damage caused by ultraviolet (UV) rays is generally believed to contribute to skin cancer, with excessive exposure to UV rays increasing the risk of both melanoma and non-melanoma skin cancer (6).

In this manuscript, we introduce the structure of the skin, the mechanism of skin aging, the composition of the microenvironment of aging skin, and the role of aging in various skin diseases, providing a new perspective for targeting aging-related mechanisms to treat skin diseases in clinical practice.

## Skin structure

**Figure 2:**
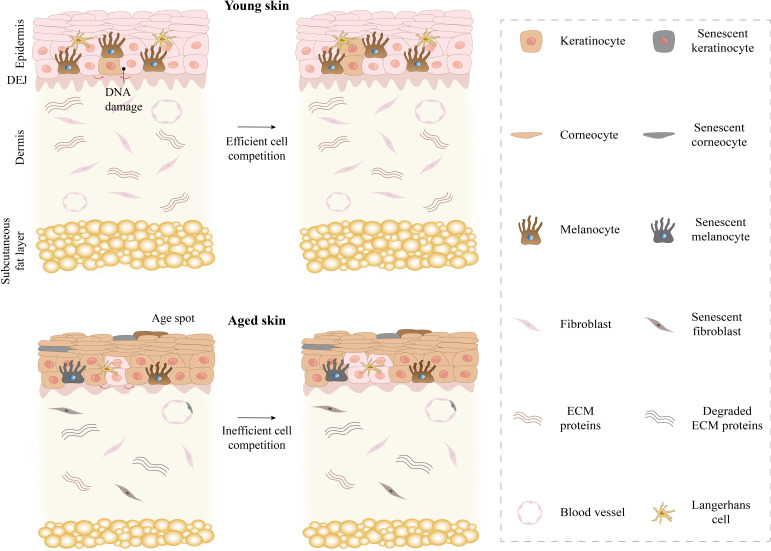
Comparison of young and aged skin. Young skin maintains its youth through effective cell competition. However, aging skin leads to skin fragility and thinning, reduced wound healing, and stem cell depletion through inefficient cell competition. ECM, extracellular matrix.

The first layer of protection against infection and injury is the skin, which is made up of various cells and structures. It serves as the largest organ in the human body and plays a crucial role in temperature regulation and vitamin production. Additionally, the sensory capabilities of the skin enable us to engage with our surroundings (7). Skin is divided into three layers from outside to inside: the epidermis, dermis and subcutaneous tissue (**Figure 2**). The epidermis and dermis are the main layers, while the subcutaneous tissue mainly stores energy and maintains warmth (8).

The epidermis consists of various layers of epithelium, interfollicular epidermis, hair follicles, sebaceous glands, and eccrine sweat glands. Its primary function is to serve as a protective barrier for the human body, guarding against physical, chemical, and biological elements. The basal layer is in the deepest layer of the epidermis and consists of a single layer of cylindrical basal cells, which are small and neatly arranged (9). The nuclei are oval, and the cytoplasm contains varying amounts of melanin granules, the content of which affects skin color. These melanin granules, produced by melanocytes between basal cells, can absorb UV rays and protect deep tissues from UV radiation damage.

Basal cells have an active proliferative capacity to produce new keratinocytes and move to the superficial layer to replenish the aging, shed keratinocytes, allowing the epidermis to achieve self-renewal (10). With the continuous proliferation of basal cells, spinous layer cells are formed, generally about 4 to 8 layers, which are polygonal cells. There are many small processes on the surface of spinous cells, and they relate to the processes between neighboring cells to form intercellular bridges. Desmosomes are also seen on the intercellular Bridges, which enhance the epidermal resistance to physical damage (11).

The superficial surface of the spinous layer is the granular layer, which consists of two to three layers of spindle cells. There were tension fibril bundles around the cytoplasm, and the membrane granules increased, which contained phospholipids and mucopolysaccharides. As the granular layer cells continue to move to the superficial layer of keratinization, mucopolysaccharides, phospholipids and other contents continue to be expelled from the membrane by particles and enter the intercellular space, which makes the adhesion between the surface layer cells firmer and can prevent the invasion of foreign materials (12). The stratum corneum, located in the most superficial layer of the epidermis, is composed of several to dozens of layers of flat, anucleated keratinocytes attached by the stratum corneum linker, which can resist many environmental stresses while acting as a permeability barrier due to the encapshement of interkeratinocyte lipids. In the thick parts of the epidermis such as the palms and soles of the feet, there is also a transparent layer between the granular layer and the stratum corneum, which acts as a barrier to prevent water loss and electrolyte passage (13).

The dermis lies beneath the epidermis and has two parts, the superficial papillary dermal layer and the deep reticular layer, which harbors blood vessels, nerves, and sensory receptors (8, 13). The cellular components of normal dermis include fibroblasts, macrophages and mast cells. In the papillary layer of the dermis, small-diameter collagen fibers are interleaved with elastic fibers consisting of elastic core proteins and microfibrous scaffolds, whereas the reticular layer is mainly composed of large-diameter collagen fibers (14). Under the epidermis, the collagen fibers near epidermal appendages and blood vessels were small and did not have a certain direction, while the collagen fibers in other parts of the dermis were combined into bundles (15). Collagen fibers are thought to be closely related to skin aging. The elastic fiber network is composed of discrete oxytalan fibers rich in fibrillin, elaunin fibers with elastin core, and elastic fibers (16, 17). In adult tissues, the microfibrous scaffold that constitutes elastic fibers is mainly composed of glycoprotein, fibrillin-1, and gives the skin elasticity (17).

Subcutaneous tissue is a loose connective tissue located under the dermis, which is fused with the dermis and has an unclear boundary (7). The primary cell types found in the subcutaneous layer are fibroblasts, adipocytes, and macrophages. These cells have distinct roles and may contribute to tissue restructuring and promotion of fat burning (18, 19). Fat cells aggregate to form primary lobules, and many primary lobules form secondary lobules, which are surrounded by fiber spacers or trabeculae. The fat septum contains blood vessels, lymphatic vessels, nerves, eccrine glands and apocrine sweat glands. Subcutaneous tissue is abundant in G protein-coupled receptors that play a crucial part in maintaining fat balance by regulating the release of lipolysis, adiponectin, and leptin (20).

## Mechanisms of skin aging

Human life will inevitably experience the process of aging, this process can lead to cells, tissues and body function decline gradually. Skin aging is characterized by increased wrinkles, senile spots, dryness, thinning, and decreased elasticity (21). The aging process of skin is not only affected by internal and time, but also by external environmental factors such as ultraviolet rays, air pollution, smoking, and malnutrition, which will accelerate skin aging and increase the number of senescent cells in the skin (22).

## Oxidative stress

Skin aging and dermal damage are attributed to oxidative stress, which is a crucial factor (23). Reactive oxygen species (ROS), generated during mitochondrial aerobic metabolism’s electron transport chain, can activate hypoxia-inducible factor 1-alpha (HIF1) and recruit immune cells to prevent skin infections. However, excessive ROS accumulation can harm DNA, lipids, and proteins, thus contributing to skin aging (24).

Oxidative stress not only raises ROS production but also accelerates intrinsic skin aging due to a decline in cellular repair capacity associated with aging (25). Only Manganese superoxide dismutase (MnSOD), the main mitochondrial neutralizer of ROS, has been shown to be crucial for the survival of aerobic life (26). Genetic variations within the human MnSOD gene play a significant role in determining the capacity of cells to counteract ROS and endure the deterioration of skin tissue. Variances in genotype might explain variations in the process of aging among individuals of a similar age. Experiments were carried out by researchers using Tet-mev-1 mice, which have mutations in their mitochondrial complex II and an excessive production of ROS. These mice displayed an abundance of apoptotic cells, resulting in decreased birth weight, delayed growth, early aging, and other pathological manifestations (27). The accumulation of mitochondrial DNA mutations in the skin, caused by aging and UV irradiation, further intensifies ROS production (28).

The impact of ROS particles relies on their dosage and duration, as well as the specific cells affected (29). Minimal levels of ROS have the potential to cause genetic mutations, moderate levels can result in replicative senescence, and elevated levels typically trigger apoptosis or necrosis (30). When the antioxidant defense is overwhelmed by ROS, the cellular environment undergoes oxidative stress, leading to consequential tissue decay and an expedited aging process. Both the direct interaction of ROS with DNA bases and the production of lipid oxidation products can cause damage to DNA. The primary form of damage is the oxidation of guanine to 8-oxoguanine (8-oxoG) by singlet oxygen (31). The nucleotide equivalent of 8-oxoG, known as 8-oxo-deoxyguanosine, leads to the creation of GC→TA, which disrupts normal cell function. These alterations are also observed in oncogenes and tumor suppressor genes, indicating their potential involvement in cancer development (32).

Furthermore, RNA is even more severely affected by ROS, resulting in reduced efficiency of translation and abnormal functioning of proteins and enzymes (33). ROS can induce damage to mitochondrial DNA (mtDNA), which undergoes mutations at a higher rate compared to genomic DNA (34). Such damage to mtDNA impairs the functionality of the electron transport chain and oxidative phosphorylation, ultimately resulting in a decrease in the production of ATP (35). Collagen and elastin can also suffer oxidative damage from ROS. ROS can oxidize amino acids, leading to alterations in the protein’s structure and conformation (36). Additionally, oxidation reactions can generate additional reactive substances that can further oxidize other biological targets. This process can induce protein cross-linking, ultimately resulting in modifications to the properties and functions of the affected proteins or enzymes (37).

Different research studies have indicated that microbial elements may play a role in responding to oxidative stress (38). In a study by Edda et al., it was demonstrated that the skin microbiota in midlife is linked to the activity of Dipeptidase D (pepD) and peptide methionine sulfoxide reductase msrA/msrB (mrs AB). PepD functions as an enzyme that breaks down dipeptides containing proline or hydroxyproline residues at the C-terminal position. Given the abundance of amino acids in collagen, PepD plays a crucial role in collagen metabolism. Meanwhile, MrsAB, produced by Campylobacterales, was observed to increase in the midlife cohort. This molecule acts as a repair enzyme for proteins that have been disabled by oxidation, serving an important role in protein restoration (39).

## Extracellular matrix degradation

Wrinkle formation, drying, impaired wound healing, skin fragility, and muscle building are consequences of the changes in the structure of collagen, elastin, and glycosaminoglycan (GAG) in the extracellular matrix (ECM) as ages (40).

**Figure 3:**
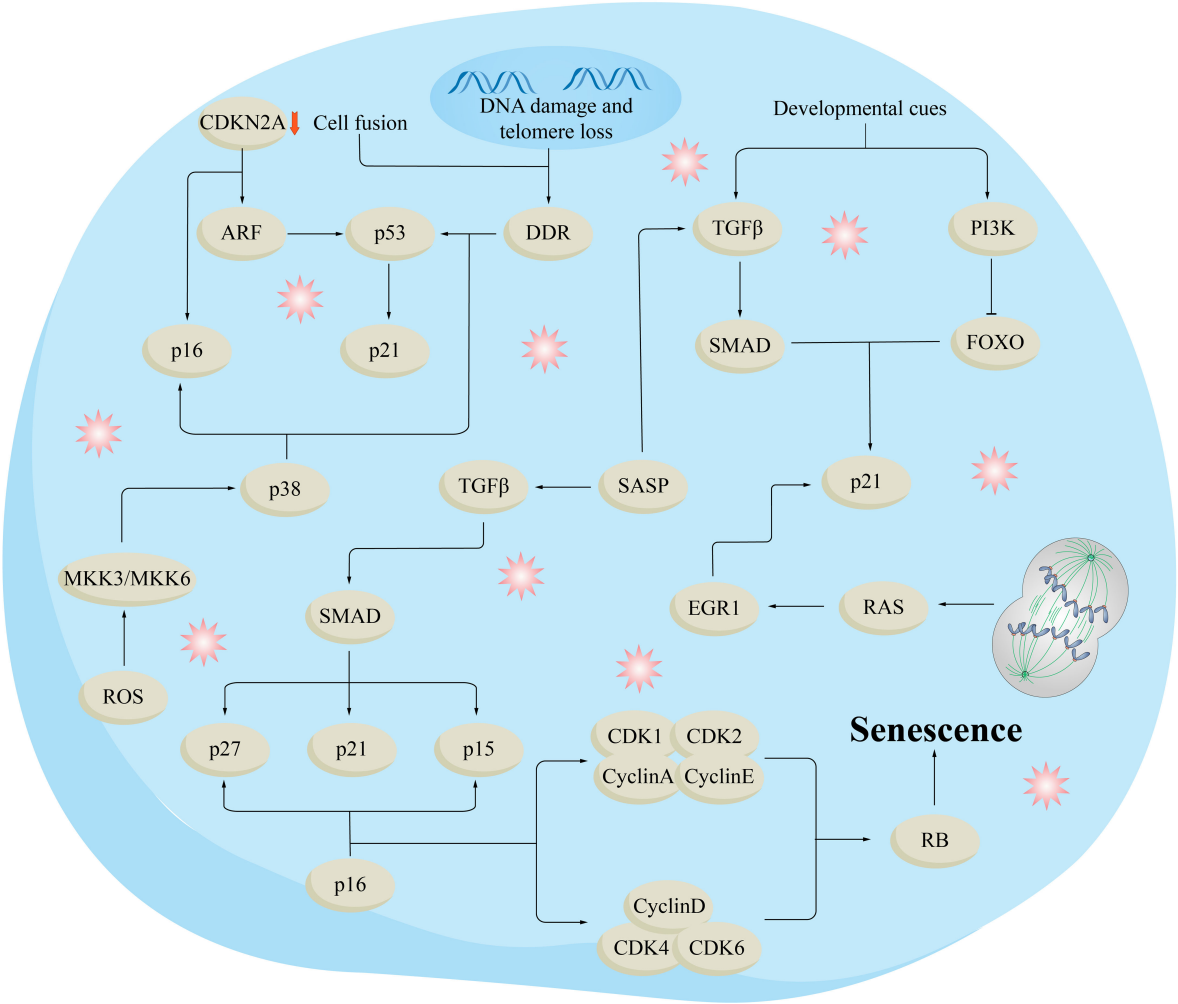
Molecular mechanisms of aging. DNA damage and telomere loss trigger the DNA-damage response (DDR), which in turn activates p53 and its downstream target p21. Different types of aging processes include the release of epigenetic constraints in the cyclin-dependent kinase inhibitor 2A (CDKN2A) gene region, which oversees generating the cell cycle inhibitor p16 and the p53 stimulator ARF. Reactive oxygen species (ROS) stimulate p16 and p53 via the kinases MKK3 (MAPKK3) and MKK6 (MAPKK6), as well as their downstream kinase p38. Oncogenic signals or the absence of tumor suppressors trigger p16 and p53, in conjunction with DDR and ARF. Transforming growth factor (TGF) plays a significant role in the senescence-linked secretory phenotype (SASP) pathway, increasing the levels of cell cycle inhibitors such as p21, p27, and p15 through the SMAD complex. Senescence induced by developmental cues involves p21 activation through the PI3K and TGF pathways. Polyploidization and cell fusion also lead to heightened p21 expression through DDR, p53, and RAS-induced activation of the transcription factor early growth response protein 1 (EGR1). These stressors and injury-causing agents trigger signaling cascades that ultimately lead to the activation of the cell cycle inhibitor and tumor suppressor RB, ultimately leading to senescence. FOXO, forkhead box protein O.

The matrix metalloproteinase (MMP) family consists of zinc-dependent endopeptidases that play a vital role in degrading the ECM. Specifically, MMP1, MMP2, MMP3, and MMP9 are responsible for this degradation process (41). Among them, MMP1 stands out as the only MMP capable of breaking down fibrillar collagen. The other types of MMPs, on the other hand, are able to further degrade collagen fragments that have already undergone degradation (42). During the process of aging, the expression of MMP increases in fibroblasts, whereas the expression of MMP inhibitors (TIMP) decreases. This is likely triggered by ROS, and increased levels of ROS activate the mitogen-activated protein kinase (MAPK) pathway (**Figure 3**). Consequently, the production of activator protein 1 (AP-1) is stimulated, leading to transcriptional regulation of MMP (43). The entire Smad signaling pathway is impaired when transforming growth factor-β (TGF-β) type II receptors are down-regulated by Ap-1, leading to a decrease in the binding of the cytokine TGF-β and subsequent reduction in the phosphorylation of Smad2 and Smad3. As a result, the transcription of the COL3A1 and COL1A1 genes, which produce type III and I collagen precursors, is reduced, ultimately leading to a decrease in the amount of type III and I collagen present in the skin (44). Moreover, as a result of aging, the DNA bases in genes that encode collagen and elastin, which are major components of the dermal matrix, undergo damage due to oxidation. Consequently, the expression of these components in aging skin decreases (45).

Another factor that can enhance MMP expression is UV radiation, possibly through the DNA damage response (DDR) pathway, which activates MAPK and transforming growth TGF-β1 (46). Following inflammation, there is infiltration of macrophages, neutrophils, and fibroblasts, which subsequently release multiple members of the elastase family, including MMP. Consequently, there is an escalation in the degradation of elastin (47).

## Inflammation

In addition to being linked to age-related cardiovascular disease, diabetes, and other chronic systemic diseases, inflammation is also connected to the aging of the skin. The cellular network of Langerhans cells (LCs) plays a crucial role in maintaining the integrity and function of the epidermal barrier. However, with aging, LCs migrate less to nearby lymph nodes, which can result in reduced immune surveillance among the elderly (48). Consequently, the compromised barrier function of the skin exposes it to external factors that can trigger chronic low-level inflammation.

In the presence of inflammation, monocytes in the skin are guided by a cytokine environment containing monocyte colony-stimulating factor to differentiate into macrophages (49). These macrophages then release MMPs and ROS in large quantities, leading to the degradation of the ECM and the development of chronic inflammation (50). The activation of protease-activated receptor 2 (PAR2) triggers an inflammatory response by phosphorylating Nuclear factor kappa-light-chain enhancer of activated B cells (NF-κB) and Forkhead box O 6 (FoxO6) through the Protein kinase B (Akt) pathway, which could serve as a crucial mechanism for ROS-induced inflammation (51). Altered production of cytokines plays a significant role in skin aging as it disrupts the balance of proinflammatory components, ultimately leading to skin inflammation.

Moreover, senescence and heightened SASP in tissues, aside from the skin, might indirectly contribute to the aging of the skin. Other internal factors include genetic defects that can lead to hereditary syndrome of premature aging, and increase the DNA damage, leading to premature aging and the increase of cell aging (52).

Notably, when bacterial microbes and their metabolites enter the circulation, they can travel throughout the body and affect distant organs and tissues, including the skin. It has been demonstrated that disorders in gut microbiota are linked to inflammatory skin diseases. As individuals age, imbalances in gut bacteria lead to the release of proinflammatory microbial products due to compromised intestinal permeability (53). These microbial metabolites contribute to damage associated with senescence-associated SASP by increasing the expression of inflammatory molecules such as TNF-α, IFN-γ, IL-1, IL-6, and MMPs, which in turn results in a persistent proinflammatory condition or inflammation (54).

## Cell senescence

Some scholars have proposed that cellular senescence may be the driver of skin aging. Intrinsic aging involves reduced cell replication, affecting keratinocytes, fibroblasts, and melanocytes in the skin. The cellular senescence process corresponds to the finite number of divisions somatic cells can undergo, which leads to gradual telomere shortening with each division. Telomeres shorten with aging, ultimately leading to extremely short telomeres, which impair the regenerative capacity of tissues and are considered one of the molecular markers of aging (55).

Telomerase, composed of a subunit called TERT and an associated RNA component known as Terc, can counterbalance the loss of telomeres by adding TTA GGG repeats to the ends of chromosomes in cells where it is typically present (56, 57). However, this alone is insufficient to counteract the telomere loss that occurs during cell division over the course of a lifetime, resulting in the shortening of telomeres with age both in the body and in laboratory settings. The DDR triggered by shortened telomeres leads to a halt in cell growth, during which the cell endeavors to mend the inflicted harm. If the DNA damage proves irreparable, it will initiate replicative senescence (58).

The effects of oxidative stress greatly affect telomeres, resulting in the buildup of DNA repair components known as telomere-associated lesions (TAFs). In experiments conducted on animals, it has been observed that the function of epidermal stem cells is impaired when telomeres undergo shortening (59). In humans, excessive exposure to UV rays causes telomeres in basal cells to shorten (60). Aging cells cannot be stimulating proliferation, it is by a variety of types of damage and stress caused by, not due to terminal differentiation, its characteristic is that through the cell cycle dependent kinase inhibitor p16 and p21CIP1, results in the stagnation of irreversible cell cycle (21).

Senescent cells can perturb their microenvironment by directly secreting senescence-related factors or by utilizing other means of communication, such as extracellular vesicles, leading to functional degradation (61). At the same time, most senescent cells also developed inflammatory secretion of SASP by paracrine effect on neighboring cells or through the endocrine function of the distance, the induced or accelerated age-related disorders (22).

Fibroblasts obtained from naturally aged skin elevate the synthesis of cysteine-rich protein 61 (CCN1), which impairs the balance of collagen and results in skin dysfunction. Furthermore, these fibroblasts also release proteins associated with skin aging (SAASP). Like the typical SASP, this protein mixture is abundant in MMP and proinflammatory substances (62). Due to the heterogeneity of senescent cells, there is no single marker that can adequately confirm their senescence status.

In recent years, a variety of indicators have been found that can be used to detect aging. Some of the biomarkers associated with aging include increased SA-β-Gal activity, elevated levels of p16INK4a and p21CIP1 (cell-cycle inhibitors), and SASP, a phenotype composed of cytokines, chemokines, metalloproteinases, growth factors, reactive metabolites, and extracellular vesicles. These changes may be one of the important causes of aging or functional impairment of tissues and organs (52, 63, 64).

## Skin aging and cell senescence

Skin aging occurs when there is an excessive buildup of senescent cells, which hampers the macrophage-dependent clearance mechanism due to SASP inhibition. Consequently, this results in a further build-up of senescent cells in the skin, causing the aging of the skin (65).

## Fibroblasts

Skin fibroblasts aging is thought to be key factors of pathological skin aging (65, 66). As fibroblasts age, they experience a decrease in the number of receptors and become less responsive to various cellular signaling molecules (67). Additionally, aged fibroblasts often have underdeveloped endoplasmic reticulum and diminished secretory activity due to a reduction in the biosynthetic machinery. This could be a significant explanation for the decline in pigment-producing cells observed in the skin of older individuals (68). There was a slower rate of proliferation of papillary fibroblasts in the dermis of older individuals, while the age did not have an effect on the proliferation of reticular fibroblasts (69). Through *in vitro* experiments, it was observed that the ECM derived from papillary fibroblasts provided better support for the longevity of the epidermis compared to ECM from reticular fibroblasts (70). Additionally, when papillary fibroblasts were aged *in vitro*, they transformed into reticular fibroblasts, indicating that an imbalance in the subtypes of fibroblasts may contribute to the aging of the skin (71).

The normal dermal fibroblasts were categorized into two groups: papillary fibroblasts and reticular fibroblasts, the common markers are COL1A1, COL1A2 et al. Papillary dermis typically contains fibroblasts that are positive for fibroblast activation protein (FAP) but negative for CD90. These fibroblasts express both PDPN and NTN1 and show resistance to lipid accumulation during adipogenic differentiation. In contrast, the FAP-CD90+ fibroblasts in the reticular dermis exhibit high levels of ACTA2, MGP, PPARγ, and CD36, indicating a greater potential for adipogenesis. This distinction between the two subgroups is crucial in delineating the characteristics of papillary and reticular fibroblasts (72, 73).

SFRP2 and FMO1 dermal fibroblasts can be categorized into two primary subgroups, which are not related to the papillary or reticular layers (74). The markers CD90 and platelet-derived growth factor receptor (PDGFR) are indicative of fibroblasts, and human dermal fibroblasts are segmented into four subsets based on their position within the dermis (75).

Senescent fibroblasts exhibit abundant SASPs, including MMP2, MMP9, IL-6, and IL-8, thereby maintaining an inflammatory phenotype (76, 77). UV exposure induced upregulation of p16INK4a, p21CIP1, and p53 in fibroblasts, presenting a senescence phenotype (78). As fibroblasts become old, they can also trigger the process of cell death in nearby cells, resulting in a reduction in the overall number of cells as one ages. These changes can lead to disruption of collagen homeostasis, delayed wound healing and increased likelihood of skin neoplasia (79). It has also been demonstrated that reduced epidermal thickness, cell damage, impaired barrier function and altered surface characteristics were found in human organ-skin models constructed with premature senescent fibroblasts (80).

## Keratinocytes

Keratinocytes are the most abundant epidermal cell type. The epidermis has a certain level of resilience to stress because it avoids the buildup of external molecular damage by shedding fully developed keratinocytes. When faced with significant oxidative stress, keratinocytes undergo apoptosis, which distinguishes them from fibroblasts (81). This shows that keratinocytes may experience minimal age-related damage accumulation (82).

## Melanocytes

The basal layer of the epidermis houses melanocytes, which have limited ability to reproduce and thus do not experience aging due to replication. Melanocyte senescence primarily occurs as a result of telomere-related aging, and skin studies on older individuals reveal specific telomere dysfunction in these cells. Interestingly, there is no significant reduction in telomere length, indicating that stress-induced changes in melanocytes can occur regardless of telomere length (83, 84).

As a person gets older, the level of p16, a biomarker for aging, rises. This increase is linked to visible changes in elastic fibers and an increase in skin wrinkles. In elderly individuals, the expression of p16 in skin melanocytes is higher due to damage to their telomeres (85). In addition, senescent melanocytes derived from senescent melanocytes can induce senescence in neighboring cells through paracrine signals involving IP10, CXCR3, and ROS (84). In areas of skin that are not exposed to sunlight, melanocytes decrease with age; however, exposed areas develop abnormal melanocyte distribution and function, resulting in pigmentation. A decrease in lamin B1 and an increase in p16INK4a were also found in melanocytic nevi (86, 87). Increased p16INK4a-positive melanocytes correlated with skin aging phenotype and wrinkle grade (85).

In addition, senescent melanocytes can induce telomere dysfunction in fibroblasts and reduce keratinocyte proliferation (84). The experiment with a 3D epidermal model demonstrated that fibroblasts and keratinocytes can both cause senescence in melanocytes. Senescent fibroblasts were found to cause skin hyperpigmentation, resulting in the senescence of melanocytes. It was observed that stress-induced activation of p53 in keratinocytes stimulated the release of chemicals that promote melanin production, potentially contributing to the senescence of melanocytes (84, 88, 89).

## Immune cells

In the epidermis, LCs primarily activate nearby immune responses by presenting antigens to T cells. They also relocate to nearby cutaneous lymph nodes to strengthen immune responses against external antigens and facilitate acceptance of self-antigens (90, 91). The number of LCS in aging skin decreases, and they also lose the ability to migrate from the epidermis when exposed to harmful stimuli because IL-1 availability in the local area decreases. As a result, the integrity of the skin barrier is compromised, and the defense against antimicrobial agents and tumor cells are weakened (92).

Most T cells found in the skin are memory T (Tm) cells, while the remaining are recycling T cells (93). Tm cells, which express CD69 and CD103 on their surfaces, exhibit enhanced immune surveillance capabilities (94). In the elderly, although the number of T cells in the body remains constant, there is a decrease in the amount of naive T cells due to thymus degeneration caused by prolonged exposure to external substances. This results in an increase in Tm cells (95). In aged skin, there is a greater ratio of CD4+ to CD8+T cells, which suggests a stronger proinflammatory phenotype comparable to young skin (96). The induction of immune tolerance type memory resident skin phenotypes near the hair follicles is associated with Foxp3+ regulatory T cells (Tregs). This can be achieved through the skin’s metabolism of short chain fatty acid sodium butyrate or increased exposure to UVB light, both of which enhance expression and proliferation of Foxp3+ Tregs (97, 98). In aging skin, there is an increased number of Tregs and the immunosuppressive receptor PD-1, which leads to the reactivation of infectious diseases by inhibiting Th1 and Th2 responses (99). Due to low stimulation efficiency, B cells in the elderly produce fewer antibodies, resulting in a reduced vaccine and antigen immune response (100). Immunosenescence with serum inflammatory cytokines such as IL - 6, IL - 8 and TNF - a higher level, at the same time accompanied by chronic low-grade systemic inflammation (101).

## Aging in skin diseases

Skin aging is a complex process that involves a variety of biochemical and physiological changes, which lead to alterations in the structure and function of the skin. In certain skin diseases, such as psoriasis, lupus erythematosus, or other autoimmune skin diseases, skin aging may manifest as an accelerated progression of the disease. For instance, the skin of psoriasis patients may age more rapidly due to ongoing inflammation, resulting in more fragile and susceptible skin. Lupus erythematosus can affect the connective tissue of the skin, leading to premature signs of aging. We have summarized the current mechanisms of aging in skin diseases, as seen in **Table 1**.

**Table 1:** Mechanisms related to aging in skin diseases.

Disease	Study subject	Mechanism	References
AD	AD patients	Filaggrin and loricrin increase with age, while Ki16 and Ki67 decline	(114)
	C57BL/6 mice	Neutrophils are required for induction of CXCL10, a ligand of the CXCR3 receptor that promotes itch via activation of sensory neurons	(245)
	C57BL/6 mice	MALT1 deficiency results in defective Treg development and CTLA-4 expression via a T Cell intrinsic mechanism	(246)
CSU	Patients	Th2-initiating cytokines (IL-33, IL-25 and TSLP) released by innate immunity contribute to the development of CSU	(247)
	Patients	Increased mast cells in the elderly have lower degranulation capacity and exhibit a consistent trypsin-chymotrypsin phenotype and simultaneous expression of granzyme b	(248)
	Patients	The association of mast cells with macrophages and VIP+ nerve fibers increased, while the interaction with blood vessels decreased	(248)
BP	Patients	Immunosenescence leads to increased serum IgG1 and IgG4 levels	(249)
	Patients	Increased drive from follicular T helper cells causing B cell activation and antibody production	(250)
Vitiligo	Patients	TNF-α can induce fibroblast senescence and is associated with depigmentation, permanent phosphorylation of p38 and ROS increase in patients with vitiligo	(251)
	C57BL6/CBA mice	Wnt/b-catenin signaling controls adipogenic cell fate within the lower dermis	(252)
	104-05n 5E5 Cells	α-MSH promotes melanin production, and levels of α-MSH are low in vitiligo	(253)
	Patients	Continuous intracellular ROS generation in the vitiligo melanocytes leads to a constitutive stimulation of antioxidant enzymes expression at the mRNA level	(254)
Melasma	Patients	Wnt5a and SFRP2, which are produced by fibroblasts, have elevated levels	(255)
	Patients	Inflammation plays a role in altering pigmentation by driving persistent inflammatory signals within the skin	(256)
	Female Patients	Keratinocytes increase ARG2 production, leading to the accumulation of intracellular melanosomes	(257)
	Patients	CDH11 causes basement membrane destruction through MMPs, which leads to pigmentation	(258)
	Patients	Sebocytes prolonge skin cell stimulation, contributing to localized dermal aging and hyperpigmentation	(259)
BCC	Patients	UV exposure increases tyrosine phosphorylation levels of -catenin, thereby controlling the signaling of Wnt/TCF in the nucleus, leading to activation of MMPs gene transcription, which is critical for tumor progression	(260)
	Patients	UVB irradiation triggers NF-kB activation	(261)
	v-rasHa transgenic Tg.AC mice	Erbb2 promotes BCC by increasing the expression of ADAM12	(262)
	K14CreER mice	The activation of the Hedgehog pathway by either loss-of-function mutations in Ptch1 gene or gain-of-function mutations in Smo4	(263)
	UW_BCC cell, Male NOD-SCID mice	Fibronectin promotes adhesion and migration of BCC cell lines through integrin α5β1-mediated phosphorylation of focal adhesion kinase	(264)
SCC	Female SKH-1 mice	The lymphatic-centered immune microenvironment underwent adaptive changes under continuous UVR via regulating YAP1/VEGFC and Piezo1	(265)
	Patients	CAFs enhance invasion by releasing chemokine ligands that alter the extracellular matrix structure	(266)
	KCs and A431 cells	UVB-induced senescent keratinocytes result in an irreversible cell cycle arrest, an increase in the proportion of senescence-associated β-galactosidase−positive cells, unrepaired DNA damage, and a long-term DNA damage response activation	(267)
CM	Patients	UV regulates skin telomerase activity	(268)
	C57BL/6 mice	MC1R is a powerful regulator of PTEN after UV exposure, and MC1R mutations caused by excessive UV exposure further promote melanoma development	(269)
	NHEMs	Diacylglycerol is present on the cell membrane of melanocytes and can be directly induced by ultraviolet light, leading to the activation of protein kinase C, which in turn regulates melanogenesis through the phosphorylation of tyrosinase	(270)
	WM35 cells	UV-induced DNA damage increases p53 levels in both keratinocytes and melanocytes, as well as melanoma cells	(271)
	A375 cells	The invasion of melanoma cells be facilitated by the action of MMPs, which modify basement membrane and ECM proteins	(272)
	UACC-903 cells	Aged fibroblasts secrete a Wnt antagonist, sFRP2, which activates a multi-step signaling cascade in melanoma cells that results in a decrease in β-catenin and MITF, and ultimately the loss of a key redox effector, APE1	(273)
	C57BL/6 mice	Treg cells and tumor cells induced responsive T cell senescence	(274)

## Aging and type 2 inflammation

**Figure 4:**
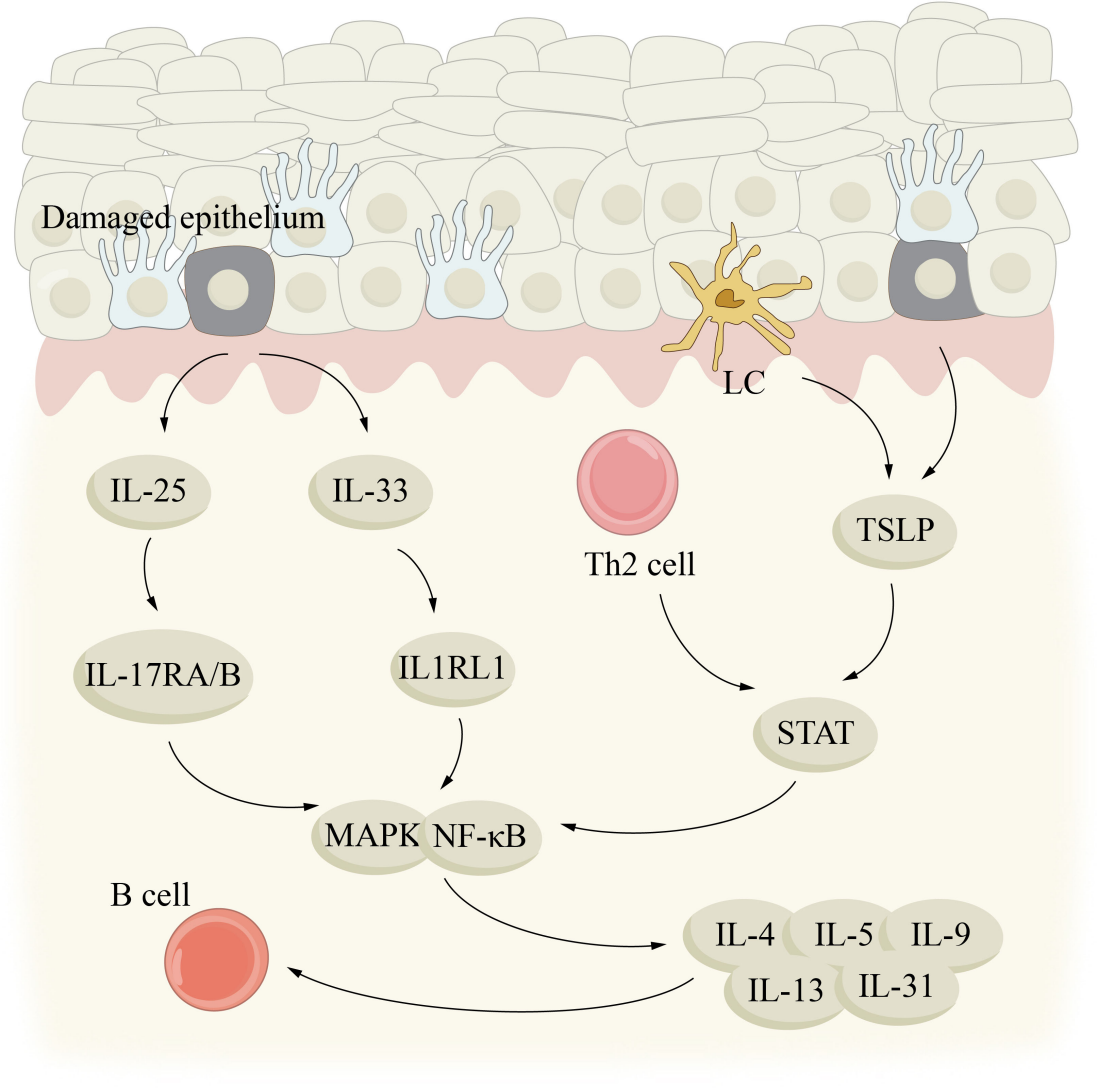
Aging and type 2 inflammation. type 2 inflammatory skin diseases, such as AD, CSU, and BP, may be driven by key cytokines IL-25, IL-33, and TSLP released by damaged epithelium and LC/DCs. IL, interleukin; LC, Langerhans cell; MAPK, mitogen-activated protein kinase; STAT, signal transducer and activator of transcription; TSLP, thymic stromal lymphopoientin.

The core of type 2 inflammation is the activation of Th2 and inherent lymphocyte2 (ILC2) pathways, and the phenotypes of type 2 inflammation usually interact and coordinate with each other in the skin and blood circulation (102). When buoyed by allergens, infection factors and toxins, IL-25, IL-33 and thymic stromal lymphatic hormone to be released in epithelial cells and Th2 cells and ILCs produce and secrete, IL-4, IL-5, IL-9 and IL-13 type 2 cytokines, thus having type 2 reaction (103, 104) (**Figure 4**). By activating B cells, IL-4 and IL-13 can induce IgE production and play a role in the migration of T cells and eosinophils into allergic inflamed tissues. In addition, they can open the epithelial tight junction barrier, which leads to leakage of the skin. In addition, cytokines can also exert anti-inflammatory effects by affecting vascular endothelial function. The promotion of epithelial cell maturation, activation, mucus production, smooth muscle contraction, and extracellular matrix production is greatly influenced by IL-13. In addition, it is also a kind of important immune adjustment factor. IL-5 plays a significant role in the maturation, recruitment and survival of eosinophils. IL-9 to participate in the formation of allergy phenotype, its action mechanism for enhancing immunoglobulin e and acidophilic granulocyte proliferation, and other aspects (102, 105–107). Type 2 inflammatory skin diseases such as AD, CSU, and BP are driven by the release of these key cytokines from damaged epithelium and LCs/DCs.

In the process of aging, the immune system of the skin will inevitably be affected, leading to its aging. The inflammation produced by skin cells during aging, characterized by low levels of proinflammatory factors, may present a complex diversity of effects. There are obvious differences in the clinical features and incidence of type 2 inflammatory skin diseases between young and old people. This difference is widely believed to be due to aging-related changes such as immunosenescence, but direct evidence is still lacking (108). The interplay among the skin microenvironment, inflammation, and skin immunosenescence in type 2 inflammatory skin diseases is intricate. Regarding inflammation, the connection between systemic immunosenescence and skin immunosenescence remains uncertain. It is crucial to identify additional intrinsic factors contributing to skin immune senescence or inflammation from a holistic viewpoint.

## AD

AD is a prevalent and persistent inflammatory skin condition that is characterized by dry skin, a rash like eczema, and intense itching. The development of AD is influenced by both allergic inflammation and deficiencies in the skin’s protective barrier (109). The common inflammatory indicator MMP-12 exhibits elevated expression levels as individuals age, whereas AD patients’ skin shows the opposite trend. As individuals get older, there is a decline in the quantity of inflammatory DCs present in AD skin, which contrasts with what is observed in normal skin. Moreover, there is a negative correlation between age and both serum IgE levels and eosinophil count, leading to a reduction in the prevalence of AD among the elderly (110). Although AD is not common in the elderly, the number of AD patients in the elderly is gradually increasing with the increasing trend of social aging. There are three different patterns of AD: late-onset AD, recurrence of AD due to a classic childhood history, and recurrence or continuation of AD in adulthood. These disorders are thought to be related to allergies. The AD of old people can be divided into two categories, endogenous and exogenous, exogenous mainly consists of indoor dust mites, pollen and food and other allergens (111). Chronic itchiness can occur in the elderly due to prolonged exposure of their skin to ultraviolet radiation and external pollution, which can cause dysfunction and a weakened immune system in the outermost layer of the skin. This is a significant reason why the elderly is more susceptible to late-onset atopic dermatitis (112). In atopic dermatitis, there is a dominance of immune responses known as Th2/Th22, and the levels of Th2/Th22 cytokines increase with age in the skin of healthy individuals (113). Furthermore, the markers associated with terminal skin cell differentiation (filaggrin and loricrin) increase with age, while the markers indicating epidermal proliferation decline (Ki16 and Ki67). This may be due to the weakening of the Th2/Th22 cell axis. The aging immune cells and immune damage caused by stromal cells are pivotal in determining the severity of atopic dermatitis in the elderly (114–116).

## CSU

CSU is defined by the presence of skin around damaged small blood vessels. It primarily consists of CD4+ lymphocytes, specifically Th2 cells, although Th1 cells and Th17 cell-derived cytokines are also found at increased levels in the blood (117). Additionally, neutrophils, eosinophils, basophils, and monocytes are also present. The process is propelled by chemokines originating from mast cells and activated endothelial cells (117). Experimental studies have shown that the number of IL-4 + and IL-5 + cells in the diseased skin of patients with CSU is increased, and the number of IL-33 +, IL-25 + and TSLP+ cells in the dermis of diseased skin is also significantly increased. This suggests that Th2-initiating cytokines (IL-33, IL-25 and TSLP) released by innate immunity could potentially contribute to the development of CSU, thus subtyping CSU as a type 2 inflammatory skin disease (118). With aging, the function of stromal cells is impaired, and the development of mast cells decreases, resulting in immunosenescence. The numbers of MC, macrophages and CD8+ T cells in the skin of the aged are increased. In the skin of older individuals, there is an increased presence of mast cells in the dermal papilla. These mast cells have lower degranulation ability and exhibit a consistent profile of tryptase-chymase phenotype and simultaneous expression of granzyme b. In addition, mast cells showed increased association with macrophages and vasoactive intestinal peptide (VIP) + nerve fibers and decreased interaction with blood vessels in aged skin (119). The most common cause of chronic urticaria(CU) was CSU. However, retrospective studies have shown that CU is uncommon in elderly patients, and elderly CU patients have a shorter course of disease than younger CU patients. The relationship between CSU and aging needs to be further studied and explored (120).

## BP

BP, known as bullous pemphigoid, is a prevalent autoimmune disorder characterized by the production of autoantibodies against skin and mucosal hemidesmosome proteins, such as BP230 and BP180. After binding to skin basement membrane zone antigens, pathogenic antibodies activate complement, induce inflammatory cell invasion, and cause inflammatory damage, which leads to the formation of subepidermal blisters (121). In the development of BP, the increased presence of Th2 cells and IL4 in the bloodstream promotes the growth of B-cells, triggers the generation of antibodies, and facilitates the switching of immunoglobulin classes. On the other hand, Th17 cells and IL-17 present in the skin activate a nearby inflammatory response mediated by neutrophils, which ultimately results in damage to the tissue (122, 123). Immunosenescence, a process of immune system dysregulation, causes the elderly to produce low-affinity immunoglobulins at an increased rate, leading to elevated serum levels of IgG1 and IgG4 (124). Additionally, chronic, sterile, low-level inflammation is common in the elderly and can be triggered by cytokines resembling those found in autoimmune diseases (125). Infections are frequent and persistent in older individuals, and in a dysregulated immune system, peptides from infectious agents can mistakenly react with similar self-peptides (126). As we age, the number of regulatory CD4 + CD25 + T cells, which help prevent autoimmune events, decreases, thus reducing the number of them may be beneficial to the development of autoimmune process (122). Previous research has identified the presence of specific anti-BP180/230 IgE antibodies in BP cases, which were found to be associated with Th2 cell-targeting cytokines IL-4/IL-13. This suggests that Th2 inhibitors may offer a safe and effective treatment option for BP patients. Overall, it is hypothesized that immune-related aging plays a significant role in the development of BP among the elderly. Nevertheless, there is a notable gap in research concerning the impact of immune-related aging and inflammation on BP (127).

## Aging and chronic pigmentary disorders

**Figure 5:**
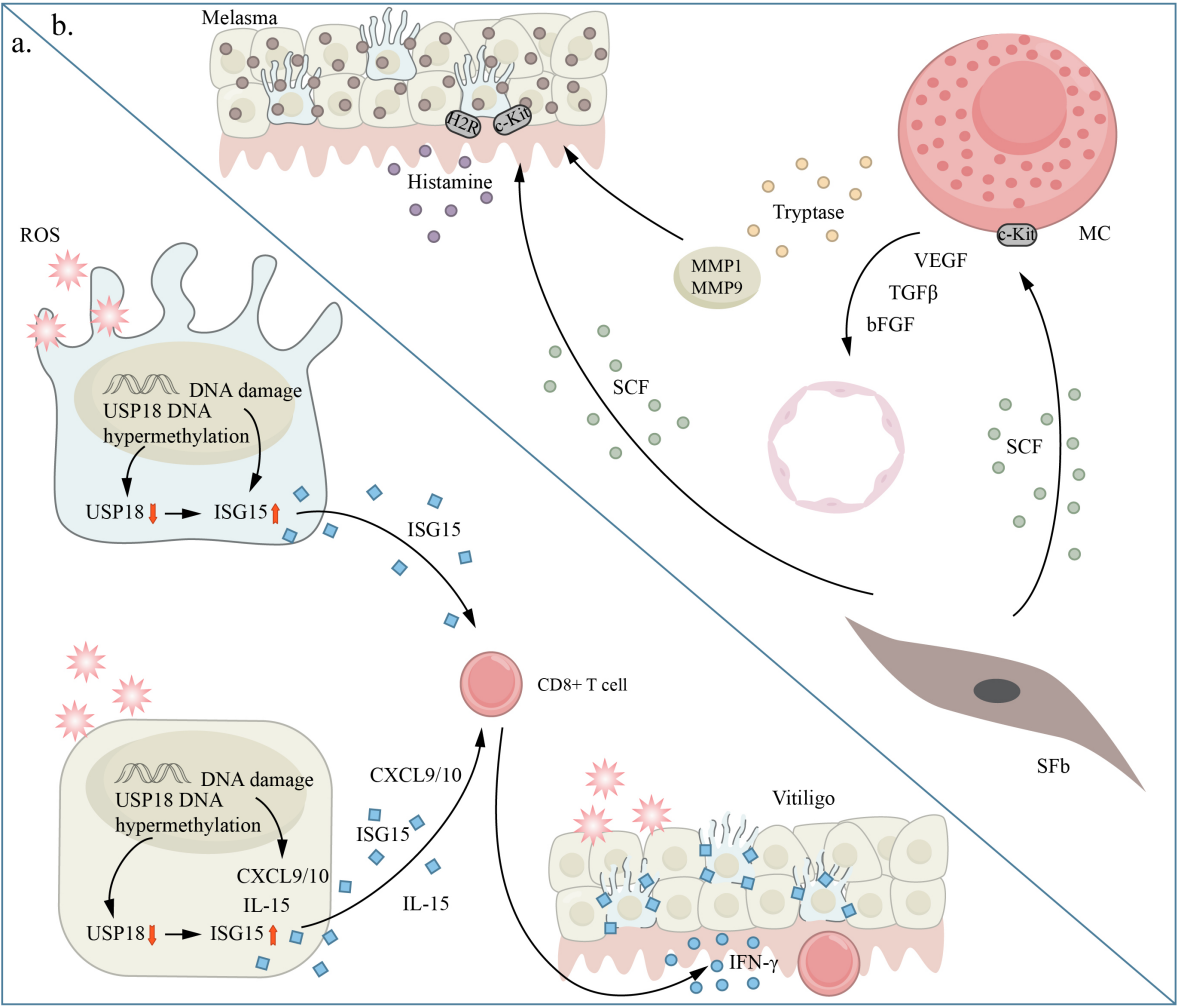
Aging and chronic pigmentary disorders. panel **(A)** shows vitiligo and panel **(B)** shows melasma. **(A)** Excessive oxidative stress is a critical factor in promoting cellular senescence through inflammation induction and immune tolerance disruption. ISG15 shows increased expression in melanocytes under excessive oxidative stress conditions. In vitiligo, ISG15 is increased in skin tissue and blood, while USP18 is decreased in vitiligo melanocytes and skin tissue. Oxidative stress causes excessive methylation of the USP18 promoter region in keratinocytes and melanocytes, a process also seen in vitiligo skin tissue. Consequently, the oxidative stress-induced hypermethylation of the USP18 promoter enhances the rise of ISG15 levels in keratinocytes and melanocytes, contributing to age-related alterations and the generation of IFN- by CD8+ T cells, which is a crucial cytokine in vitiligo pathogenesis. **(B)** Histamine directly enhances melanin production by interacting with H2 receptors (H2Rs) on melanocytes (Mels). In melasma, SCF is upregulated and affects the survival, migration, and activation of melanocytes. This is achieved through its binding to the c-KIT receptor, which triggers melanin production in Mel cells. bFGF, b fibroblast growth factor; CXCL, (C-X-C motif) chemokine ligand; IL, interleukin; MC, mast cell; MMP matrix metalloproteinase; SCF, stem cell factor; SFb, Senescent fibroblast; VEGF, vascular endothelial growth factor.

Previous research has indicated that premature aging is linked to vitiligo and melasma (**Figure 5**). These two contrasting skin pigmentation disorders demonstrate that faulty functioning is not limited to melanocytes alone. The regulation of skin pigmentation is influenced by both dermal and epidermal cells through intricate intercellular communication. The aging process primarily affects the dermal layer and its paracrine function. This may be connected to the absence of melanocytes in vitiligo lesions and the heightened activity of melanocytes in melasma pigmentation spots.

## Vitiligo

Vitiligo is the leading cause of hypopigmentation globally, affecting approximately 1% of the population. This acquired pigmentary disorder is widely prevalent. Vitiligo leads to noticeable areas of depigmentation on the skin, which can appear anywhere on the body. The condition has a significant impact on the mental well-being of patients and adversely affects their overall quality of life (128, 129).

In Webb et al. ‘s study, it was found that long-term use of TNF-α caused fibroblast senescence (130). TNF-α is associated with depigmentation, permanent phosphorylation of p38, and ROS increase in vitiligo (131). The diversity of individual dermal compartments in human skin is constituted by the differential expression of Wnt/β-catenin pathway genes (such as Wnt5a,Lef1,Tcf4,and DKK1) between the upper and lower layers of the dermis (132). Secretion factors that regulate the Wnt/β-catenin pathway play a role in both melanocyte differentiation and the mobilization of melanocyte stem cells. It has been shown through experiments that vitiligo melanocytes experience oxidative stress (133). The Wnt/β beta-catenin pathway is involved in combating oxidative stress (134). Dysregulation of the Wnt/β-catenin pathway has been observed in vitiligo disease, which may explain the heightened vulnerability of vitiligo cells due to a decreased protective ability of this pathway (135). Moreover, the inhibition of the Wnt pathway is associated with the onset of cell senescence. Thus, the increased expression of DKK1 in the dermis at the site of vitiligo lesions is linked to the aging characteristics displayed by melanocytes.

IGFBP7 is a member of the SASP factor family, and it can suppress the signaling process through the IGF-1 receptor (IGFIR). This receptor is believed to be the primary mechanism responsible for aging in melanocyte lineage, and IGFBP7’s secretion may have a significant impact on the development of vitiligo (136). Vitiligo affected skin exhibits similarities to the aging of skin, including the degeneration of sweat and sebaceous glands (137). As individuals age, there is an increase in the production of α-melanocyte stimulating hormone (α-MSH) in the outer layer of the skin known as the epidermis. However, there is a significant decrease in the number of receptors for α-MSH. This indicates that α-MSH-mediated signaling is likely involved in the process of skin aging (138). Additionally, α-MSH plays a protective role against damage to mtDNA in keratinocytes, while it may actually contribute to mtDNA damage in melanocytes by promoting melanin production. Interestingly, there is a decrease in α-MSH levels in both the epidermal and melanocyte cells of individuals with vitiligo, suggesting a potential connection between lower levels of α-MSH and the aging characteristics observed in vitiligo cells (139, 140).

## Melasma

Melasma is a common acquired hyperpigmentation disorder, which occurs mostly in middle-aged women with a chronic course and is prone to relapse. The rash is mostly symmetrical distribution, irregular border, and has a lot to do with sun exposure, usually aggravated in spring and summer, autumn and winter to reduce (141).

It is generally believed that chloasma has much to do with sun exposure, but UV is not always necessary for the development of chloasma and there are other factors involved in promoting its development (142). Studies have shown that melasma is closely related to cellular senescence. The presence of age-related spots may be attributed to local changes in fibroblast architecture, where the number and growth of fibroblasts decrease along with their secretory activity as one ages (143). Elevated levels of Wnt5a and sFRP2, which are substances produced by fibroblasts, appear to be connected to the development of melasma (144).

In melasma, the dermal-epidermal network is responsible for regulating the imbalance in skin pigmentation. The activation of the P38 MAPK pathway, which plays a role in stress responses in various skin conditions and photoaging, is involved in this process. Analysis of senescent cells damaged in the upper dermis revealed the presence of p38 MAPK, highlighting the significance of senescence in the continual pigmentation seen in melasma. Many skin diseases, including skin aging, are characterized by a common occurrence of chronic low-grade inflammation (145, 146). As individuals age, there is an upregulation of IL-6, which is the most vital cytokine in the SASP and is associated with the aging process of human keratinocytes, melanocytes, and fibroblasts. This upregulation reflects the persistent inflammatory state that accompanies aging (147, 148). Inflammation plays a role in altering pigmentation by driving persistent inflammatory signals within the skin (149). Patients with chloasma exhibit significantly higher permeability of CD4+ T cells, CD68+ macrophages, and mast cells in skin lesions compared to healthy individuals (150). Additionally, there is an overexpression of inflammatory mediators such as iNOS, Cox-2, and IL-17 in chloasma (151).

The enzyme arginase (ARG2) plays a significant role in the aging of cells. In individuals with melasma, keratinocytes experience an increased production of ARG2, resulting in the buildup of melanosomes within these cells. Furthermore, keratinocytes affected by chloasma exhibit characteristics reminiscent of the aging process, such as larger nuclei, irregular shapes, and variations in chromatin (152). Senescent cells secrete IGFBS, which can further stimulate the abnormal accumulation of cells through autocrine and paracrine mechanisms. The expression of IGFBP3 is elevated in melasma at the level of mRNA (144).

Excessive exposure to UV rays can result in an increased expression of MMP2 and MMP9, which could be responsible for the dysfunction of the basement membrane in melasma (153). Interestingly, some researchers have suggested that this dysfunction is not caused by UV irradiation but rather by cadherin-11 (CDH11), which leads to the destruction of the basement membrane through MMPs and subsequently causes hyperpigmentation. CDH11, a cadherin that enhances melanogenesis through direct interaction between fibroblasts and melanocytes, was found to be upregulated in areas affected by melasma (3, 142).

## Aging and skin cancer

**Figure 6:**
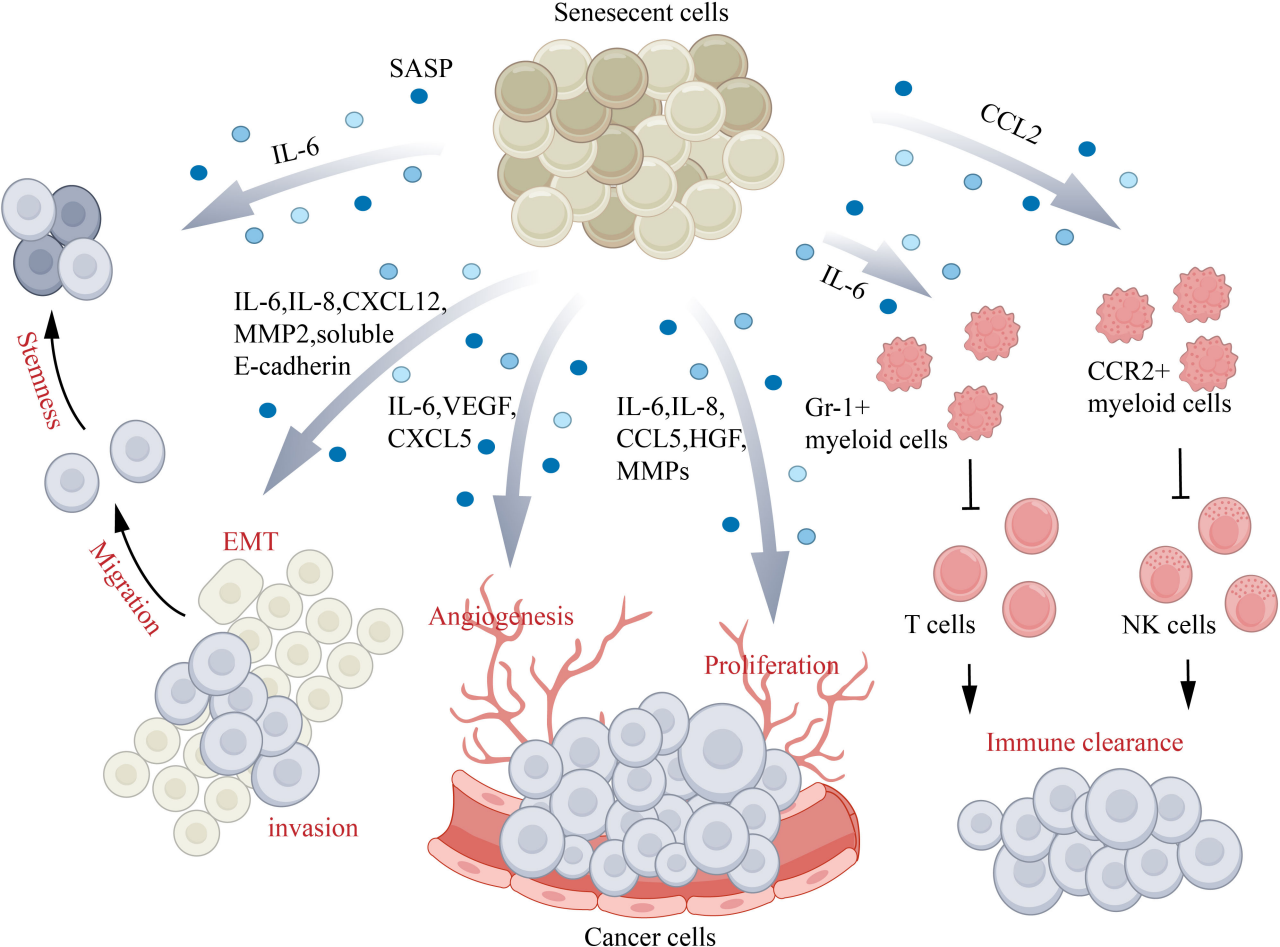
Aging and skin cancer. Senescence-associated secretory phenotype (SASP) factors play a role in different aspects of cancer progression: IL-6 and IL-8 assist in promoting EMT, IL-6–IL-6R–STAT3/CCL5–CCR5–c-Myc/HGF contribute to cancer cell proliferation, CXCL5 and VEGF aid in angiogenesis, and CXCL12–CXCR4/IL-6/IL-8/eotaxin/CCL5/MMPs promote cancer cell invasion and migration. Additionally, IL-6 inhibits the CD45+CD3+ T cell-mediated immune clearance of cancer cells by activating Gr-1+ myeloid cells, while CCL2 inhibits natural killer (NK) cell-mediated immune clearance of cancer cells by activating CCR2+ myeloid cells. CCL, (C-C motif) chemokine ligand; CCR, (C-C motif) chemokine receptor; CXCL, (C-X-C motif) chemokine ligand; EMT, epithelial–mesenchymal transition; HGF, hepatocyte growth factor; IL, interleukin; MMP, matrix metalloproteinase; VEGF, vascular endothelial growth factor.

Aging is a tumor suppressor reaction, when the cancer stress stimulation, p53, and p16 tumor suppressor proteins control ways are established, so as to prevent stress cells in cell cycle progression and ultimately to the development of malignant tumor (154). However, while young senescent cells can protect organisms from cancer, but with the passage of time, the aging cells can evade the immune system and accumulation in the organization, secretion of proinflammatory cytokines, growth factors and protease, which establish a microenvironment that promote cancer (155) (**Figure 6**). Senescence plays a complex role in cancer biology as it both hinders the unchecked growth of damaged cells and supports their removal through paracrine signaling. However, the build-up of damage and the removal of damaged cells can also raise the risk of developing cancer. Presently, there is no definitive proof that aging skin creates a conducive environment for the growth of tumor cells. Previous experiments using two senescence inducers (TPA and DOXO) and p16-3MR mice have shown that the progression of skin cancer is facilitated by cellular senescence (156). The connection between the presence of long-standing senescent cells and the proliferation of tumor cells is well-established. The controlled senescence of fibroblasts, when occurring in a controlled environment, facilitates the growth of precancerous epithelium and gives rise to tumor cells. This process, observed in mice, ultimately leads to immune impairment (4). As people age, the structure of their skin undergoes transformations such as the thinning of the outer layer and underlying layer, increased loss of moisture, and deterioration of collagen and elastin. Moreover, there is a decline in the immune components of the skin, including a decrease in Langerhans cells and antigen-specific immune response reduction. On the other hand, the presence of regulatory groups like Foxp3+ regulatory T cells increases. These alterations in the immune system of older individuals result in a weakened barrier function, making them more vulnerable to the development of cancer (5).

The exact influence of aging on UV radiation-induced skin cancer remains uncertain. Nevertheless, it is notable that skin cancer prominently emerges during middle and later stages of life. When exposed to the same amount of sunlight, individuals who are 60 years and older face a higher susceptibility to developing skin cancer compared to those who are younger than 60. Nonetheless, the primary risk factor in the development of these cancers is exposure to UV radiation. Consequently, the concept of “photoaging” arises, which generally encompasses changes in the characteristics of “skin aging,” such as wrinkles, pigmentation, telangiectasia, atrophy, fragility, ecchymosis, primarily caused by damage from sun exposure (6). DNA damage caused by UV exposure is commonly thought to cause skin cancer. The occurrence of skin cancer is significantly promoted by the inflammatory response of immune cells in the tumor microenvironment, as well as the activation of multiple oncogenes and the inactivation of tumor suppressor genes caused by UV exposure, resulting in genetic mutations (157). The expression of MMPs in human skin is increased by ultraviolet radiation. MMPs can break down proteins in the ECM, which results in photoaging. This breakdown of the ECM enables tumor cells to invade the basement membrane and the surrounding fibrous collagen stroma. Furthermore, MMPs contribute to angiogenesis, promoting the growth and migration of cancer cells (158). Excessive exposure to UV radiation is a significant environmental hazard that increases the risk of melanoma and non-melanoma skin cancers. Non-melanoma skin cancers consist of two types: basal cell carcinoma (BCC) and squamous cell carcinoma (SCC), with BCC accounting for approximately 4/5 of cases and SCC accounting for approximately 1/5 of cases (159, 160).

## BCC

BCC affects a growing number of older adults in America each year, making it the most prevalent cancer globally with millions of cases (161). In one study, patients with BCC located in the perianal and genital areas were 73 years old on average, 36% had a history of skin cancer at sun-exposed sites, and 21% likely had a related disease. Therefore, advancing age and local trauma may lead to the onset of BCC in the perianal and genital skin (162). The TP53 gene, known as a tumor suppressor gene, plays a role in triggering cell cycle arrest and programmed cell death (163).

In BCC, TP53 mutations primarily occur as C-T transitions, and there is a prominent occurrence of the double base change from CC to TT, suggesting that these alterations are caused by exposure to UVR (164). Previous research has observed a high frequency of mutations in TERT promoters in BCC, and many of these mutations exhibit UV signatures (165, 166). The exposure to UV rays increases the level of tyrosine phosphorylation of -catenin, which in turn controls the signaling of Wnt/T-cell transcription factor (TCF) in the nucleus (167). This ultimately leads to the activation of gene transcription for MMPs, which are essential for the progression of tumors. Inflammation is found to be associated with the presence of MMP-13, MMP-1, and MMP-9 in the vicinity of BCC, suggesting that inflammation plays a significant role in regulating the advancement of tumors (160). MMP-13 is expressed by both tumor cells and the surrounding stromal cells of epithelial tumors. It is responsible for the breakdown of the extracellular matrix (ECM) and is linked to the transformation of skin carcinomas into a malignant state (168). MMP-1 contributes to the early stages of tumor proliferation by cutting ECM proteins and activating growth factors that subsequently stimulate the growth of cancer cells (169). MMP-9 exhibits proteolytic activity on components of the basement membrane, particularly type IV collagen (159). Nuclear factor-kappa B (NF-kB) has a significant role in inflammation and cell growth, which are both connected to the development of tumors (170, 171).

Additionally, studies have revealed that NF-kB p65 could potentially enhance the progression of remarkably aggressive BCC. It is believed that exposure to UVB radiation triggers the activation of NF-kB, subsequently leading to the formation of skin cancer (172). In a study, Johnson and Wulff discovered that keratinocytes release HMGB1 when exposed to UVR in laboratory settings. This finding suggests that, with repeated and prolonged UVR exposure, HMGB1 could potentially be present in skin tumors. Just like NF-kB, HMGB1 released from tumor cells undergoing necrosis is notably found in areas outside of BCC cells (173, 174). Research has indicated that Toll-like receptors (TLR) play a role in enhancing the body’s defense mechanisms and genes responsible for DNA repair. TLRs are also involved in promoting functional DNA repair, repairing DNA damage caused by UVB radiation, and stimulating TLR2,3,4,7,8, and 9. Among these receptors, TLR7 is specifically found on the membrane of endosomes and is prominently expressed in BCC (175). Erbb2, also known as human epithelial growth factor receptor 2 (HER2)/neu, functions as a proto-oncogene. Its role is to promote the development of skin cancer by increasing the level of ADAM12 expression. The activation of ADAM12 is triggered by exposure to UVR (176, 177). The presence of NOD-like receptor thermal protein domain associated protein 3 (NLRP3) has been identified in human BCC cells, which contributes to the inflammatory response in BCC. The exposure to UVB radiation can hinder the movement of Ca2+ by reducing the expression of sarco/endoplasmic reticulum Ca2+-ATPase (SERCA2), thus facilitating the activation of NLRP3 inflammasome (157).

## SCC

SCC accounts for approximately 1/5 all cutaneous malignancies and is characterized trolled growth of keratinocytes in the epidermis (178, 179). The incidence of SCC is twice as high in men as it is in women, and the average age at which SCC is diagnosed is 70 years old (180). Factors that increase the risk of cutaneous SCC include age, exposure to UV radiation, race, skin type, and weakened immune system (181). UV radiation is widely recognized as the primary factor that poses a danger to the development of SCC (178, 179).

When collagen VI, a crucial part of the fibrils that connect the basement membrane to the dermis, is lost, it enhances the infiltration of keratinocytes and the transition from epithelial to mesenchymal cells (182). The cleavage of collagen IV by MMP-2 and MMP-9, which are prevalent in the basement membrane, plays a crucial role in the invasion and spread of SCC. MMP-2, produced by fibroblasts, is expressed in the initial stages of tumor formation, initiating tumor growth (158). On the other hand, MMP-9, derived from inflammatory cells like neutrophils, mast cells, and macrophages, promotes tumor invasion and angiogenesis by facilitating the release of TGF- and VEGF (183, 184). These enzymes, MMP-2 and MMP-9, regulate the activity of numerous growth factors and cytokines, impacting immune responses and angiogenesis. Consequently, they contribute to the proliferation and perpetuation of both primary and metastatic tumors (185, 186). After the basement membrane is destroyed by tumor cells, cancer - associated (myo -) fibroblast (CAF) abound in the tumor environment (187). CAFs enhance invasion by releasing chemokine ligands that alter the extracellular matrix structure (188). The stroma of SCC exhibits higher levels of MMP-2 expression in comparison to BCC, potentially explaining the varying invasion patterns observed in these two types of tumors (189). Studies have shown that the skin sunburned by UV radiation has a higher level of telomerase activation, so UV can regulate skin telomerase activity. In addition, telomerase activation was observed in the skin of patients with aggressive SCC (190, 191).

## CM

Genetic, epigenetic, and allogeneic alterations of melanocytes (MCs) are responsible for the development of cutaneous melanoma. Despite representing only 10% of all skin cancers, melanoma has a significantly high mortality rate, contributing to approximately 80% of deaths related to skin cancer (192).

Research on the spread of cutaneous melanoma (CM) has indicated that several environmental factors, such as ultraviolet radiation, alcohol intake, obesity, heavy metals, and certain pesticides, are linked to its occurrence. The main external cause of melanoma formation is ultraviolet radiation, with both UVA (315-400 nm) and UVB (280-315 nm) being capable of promoting its development. The majority of melanomas exhibit numerous somatic mutations, and most of these mutations are attributed to UV exposure, with 60% presenting as C→T changes and 5% as CC→TT changes at the bipyrimidine site (193). Exposure to UVB radiation in mice expressing CDK4R24C, a cancer-causing form of CDK4, accelerates the formation and growth of melanoma tumors (194). In a high-risk cancer family, a hereditary nonsense mutation in BAP1 (Y646X) and exposure to asbestos and UV radiation in the environment were found to contribute significantly to the increased occurrence of cutaneous melanoma (195). It has been scientifically proven that melanocortin 1 receptors (MC1R) is a powerful regulator of the phosphatase gene (PTEN) following UV exposure, and the mutation of MC1R resulting from excessive UV exposure further enhances the development of melanoma (196). Diacylglycerol, found in the membrane of melanocytes, can be directly induced the formation by ultraviolet rays. This leads to the activation of protein kinase C, which in turn regulates melanogenesis through the phosphorylation of tyrosinase (197, 198). Moreover, UV-induced DNA damage increases p53 levels in both keratinocytes and melanocytes, as well as melanoma cells (199, 200). This results in preferential transactivation of pro-opiomelanocortin (POMC) expression in epidermal keratinocytes. POMC is then cleaved to produce α-MSH, which binds to MC1R on the melanocyte membrane (201). This stimulates the production of a circular APM, which activates the transcription of melanases TYR and DCT, induced by the microphthalmic-associated transcription factor (MITF) (202).

Similar to non-melanoma skin cancer, the invasion of melanoma cells can be facilitated by the action of MMPs, which modify basement membrane and ECM proteins (203). To support tumor growth, melanoma cells release MMP-7, MMP-15, and MMP-16, while fibroblasts secrete MMP-2 and MMP-14 (158). Moreover, MMP-14 not only promotes growth but also stimulates tumor angiogenesis by increasing the production of VEGF. MMP-9 originating from inflammatory cells contributes to the occurrence and progression of tumors, along with tumor angiogenesis (204, 205). Both MMP-2 and MMP-9 are considered essential for the advancement of melanoma cells. Metastatic melanoma characteristically expresses MMP-3, which activates precursor MMP and is linked to reduced disease-free survival (206). The presence of MMP-12 is associated with melanoma cell invasion, lymph node and tumor metastasis, as well as prognosis in melanoma patients (207).

## Treatment

Maintenance treatment for AD involves the frequent use of emollients and daily bathing with cleansers that do not contain soap (208, 209). When there is a flare-up of AD, the treatment modality of choice is topical corticosteroids. Topical calcineurin inhibitors, such as pimecrolimus and tacrolimus, can also be used in combination with topical corticosteroids (210). UV phototherapy is a safe and effective option for treating moderate to severe cases of atopic dermatitis. Secondary skin infections can be effectively treated with anti-staphylococcal antibiotics (208). Although newer medications like crisaborole and dupilumab have shown effectiveness in treating AD, their high cost makes them inaccessible to most patients at present (211, 212). The communication between inflammatory COL6A5+ COL18A1+ fibroblasts subpopulation producing CCL19 and T cells and LAMP31 DCs through CCR7 is essential in controlling the organization and movement of lymphoid cells in AD lesions. Modulating the function of a subset of fibroblasts may be a good therapeutic target for AD (73, 213).

International guidelines in 2021 require the treatment of CSU to be upgraded from second-generation H1-antihistamines (sgAHs) to omalizumab and cyclosporine (214). Cupping therapy may enhance efficacy when used as an adjunctive therapy but should be used with caution (215). Furthermore, research has demonstrated that Remibrutinib exhibits a notable level of effectiveness when used to treat CSU, exhibiting a speedy onset of action and a favorable safety profile (216). Researchers are currently studying new therapies specifically designed for the treatment of CSU, including ligelizumab, fenebrutinib, and remibrutinib, which target anti-ige, as well as dupilumab, which targets anti-IL-4RR (217).

The treatment for BP varies depending on the severity of the disease and the overall health of the patient. Topical glucocorticoids have become the preferred first-line treatment for BP, but there is debate about their long-term feasibility. Dupilumab combined with methylprednisolone and azathioprine demonstrated benefits in managing the progression of the disease and expediting the decrease in glucocorticoid dosage (218). BP often leads to complications like severe itching and the presence of blisters and skin lesions, which significantly impact quality of life. Therefore, it is crucial to manage itching and minimize potential side effects. The recommended treatment duration is typically 6-12 months, unless the patient is unresponsive to corticosteroids. In such cases, a maintenance period may be necessary, which involves low-dose oral pbluenisone or topical clobetasol propionate for 1-6 months after the active disease is resolved (219–221). There are already biologics available in the market that can selectively block the formation of autoantibodies, the inflammatory cascade, or both. These biologics could potentially serve as alternative therapies to combat BP in the future (222). Furthermore, the latest research indicates that when treating elderly individuals, managing BP should primarily focus on addressing the negative impacts of aging on the skin. This can be achieved by enhancing skin barrier function and promoting skin balance through regular application of moisturizers and protection from sunlight. This approach is crucial because age-related health conditions in the elderly often limit the effectiveness of immune-suppressing treatments available (223).

A very small number of patients with vitiligo may regress spontaneously, and most patients require treatment to achieve good control of vitiligo (224). Successful repigmentation of vitiligo requires suppression of autoimmunity and promotion of melanocyte regeneration. In advanced vitiligo, topical corticosteroids and calcineurin inhibitors can be used to promote repigmentation of vitiligo (225). PUVA treatment, which utilizes UVA to transform psoralen compounds into oxidative chemical products that react with DNA, is highly effective in treating vitiligo. This treatment not only inhibits autoimmunity but also boosts the growth and pigmentation of melanocytes (226). Recent research has revealed that narrow-band UVB phototherapy can produce superior repigmentation results in vitiligo lesions compared to PUVA, while also causing fewer side effects. Therefore, photosensitive drugs (such as psoraleae tincture) combined with sunlight or narrow-band UVB irradiation can be used for stable vitiligo (227–229). At present, the treatment of vitiligo is still not ideal, all treatments only show effect in the short term, and the recurrence rate of patients within 1 year after stopping treatment is almost half (230). As a result, although safer regimens have replaced many previous treatments in modern times, efforts to explore more efficient targeted therapies are ongoing. A recent discovery revealed that a specific group of fibroblasts that respond to IFNγ are specially tasked with attracting and stimulating CD8+ cytotoxic T cells by releasing chemokines like CXCL9 and CXCL10 (231, 232). On the other hand, type 2 cytokines like CCL2 and CCL8 are crucial for shaping the environment conducive to vitiligo development (233). In an experimental setting conducted in a laboratory, it was shown that stimulating IFNγ led to elevated expression of CCL2 and CCL8 by activating the JAK-STAT pathway in vitiligo fibroblasts (234). Furthermore, studies have shown that Lycium barbarum polysaccharide (LBP) can shield keratinocytes and fibroblasts from oxidative stress. This safeguarding effect is likely connected to the control of the STAT3-Hsp70-CXCL9/CXCL10 pathway (73, 235). At the same time, patients should also pay attention to mental relaxation, regular life, balanced diet and avoid exposure to the sun in daily life (224).

Melasma is difficult to treat with a high recurrence rate, and there is no universally effective treatment. Triple therapy, a combination of benzo phenol, retinoic acid, and corticosteroids, is considered the most effective treatment (141). Benzo phenol interferes with tyrosinase activity, retinoic acid can combat the aging process, and corticosteroids are able to relieve mild inflammation caused by light damage (236). Glycolic acid chemical exfoliation is also effective in the treatment of melasma, but this method has a certain degree of skin irritation and can lead to post-inflammatory hyperpigmentation. Similarly, laser therapy and intense pulsed light are very beneficial, but both require attention to adverse effects (237). However, the recurrence rate remains high for all treatment modalities. Patients should avoid inducing external stimuli and UV irradiation as much as possible.

Surgical resection is the main treatment for skin cancer. In cases where surgical resection is not feasible for NMSC, electrode drying and curettage or diathermy can be effective, while liquid nitrogen is effective for superficial lesions. Vismodegib, a hedgehog (Hh) pathway inhibitor, has recently been approved for the treatment of advanced or metastatic BCC that is refractory to other treatments (238). SCC patients often have overexpression of the epidermal growth factor receptor (EGFR) (239). Cetuximab, an inhibitor of EGFR, has demonstrated effectiveness in treating locally advanced, metastatic, or recurrent head and neck SCC (239). Alongside EGFR inhibitors, the efficacy of antibodies targeting vascular endothelial growth factor receptors and other tyrosine kinase inhibitors in treating SCC is currently being investigated. Furthermore, several studies and reports have indicated the benefit of capecitabine in treating advanced or recurrent head and neck SCC (240). For superficial BCC that is not suitable for surgery or other treatments, imiquimod can be used, while 5-fluorouracil (5-FU) is used for SCC treatment. Radiotherapy is primarily used to control marginal lesions or treat very large or poorly positioned lesions, and it has remarkable curative effects. However, caution should be exercised when considering radiotherapy for elderly patients (241). Photodynamic therapy (PDT) is an option for treating superficial BCC by applying a photosensitive cream and exposing it to bright light. PDT, either alone or in combination with local immunomodulators, is also effective for treating NMSC and precancerous lesions (242). The use of non-surgical agents like imiquimod, interferon (IFN), and 5-FU has significantly reduced morbidity and mortality while improving quality of life.

For CM, surgical resection with a 5 mm margin is necessary, with larger margins required for treating large or poorly defined lesions. When dealing with melanomas appearing on UV-damaged skin with unclear boundaries, Mohs surgery may be the best option (243). IFN therapy can be used as adjuvant therapy for stage II and III melanomas and as an alternative when surgical options are not available. Systemic chemotherapy (dacarbazine, temozolomide, or carboplatin/paclitaxel) plays a crucial role for some patients with metastatic disease. Surgery is no longer effective in treating advanced cutaneous melanoma. In the past, chemotherapy drugs were the main treatment option. However, the emergence of immunotherapy, targeted therapy vaccines, small molecule therapies, and combination therapies in recent years have offered hope in terms of both effectiveness and safety for treating melanoma (244). Additionally, targeted therapies such as BRAF inhibitors (e.g., Vemurafenib) for patients with B-RAF mutations and CTLA-4 antibody ipilimumab provide new therapeutic possibilities alongside conventional chemotherapy (241).

In addition to AD and vitiligo, other skin diseases may also be related to fibroblast subsets, and regulating the function of fibroblast subsets provides new ideas and new targets for the treatment of some skin diseases.

## Conclusion

Skin aging is associated with many skin diseases. This article specifically describes the correlation between type 2 inflammation, chronic pigmentary disorders, skin cancer and skin aging, and proposes some treatment methods.

However, the exact mechanism of action of these diseases and skin aging remains to be elucidated, and some conclusions are controversial and need further research to determine. For example, the negative correlation between age and Th2/Th22-related cytokine levels in the skin of elderly AD patients are disputed due to the excessive secretion of multiple cytokines in the skin of healthy elderly individuals. Aging both impedes the uncontrolled growth of damaged cells and supports their removal by paracrine signals. However, the accumulation of damage and the clearance of damaged cells also increase the risk of cancer. In the future, more animal experiments and clinical experiments can be carried out to verify whether skin aging causes or aggravates these skin diseases.

Although the exact mechanism is still unclear, some possible mechanisms proposed in this manuscript provide new ideas for clinical treatment and new drug development. At the same time, more attention should be paid to skin diseases in the elderly to achieve early detection, early diagnosis and early treatment.
